# Supplementary material for: Low potential enzymatic hydride transfer via highly cooperative and inversely functionalized flavin cofactors
Source: Nat Commun. 2019 May 6;10:2074. doi: 10.1038/s41467-019-10078-3 (PMC6502838; doi:10.1038/s41467-019-10078-3)
Supplement: Supplementary file 1 — Supplementary Information [file 41467_2019_10078_MOESM1_ESM.pdf]

Supplementary information

Low potential enzymatic hydride transfer via highly cooperative and inversely functionalized flavin cofactors

Willistein et al.

Supplementary Figures

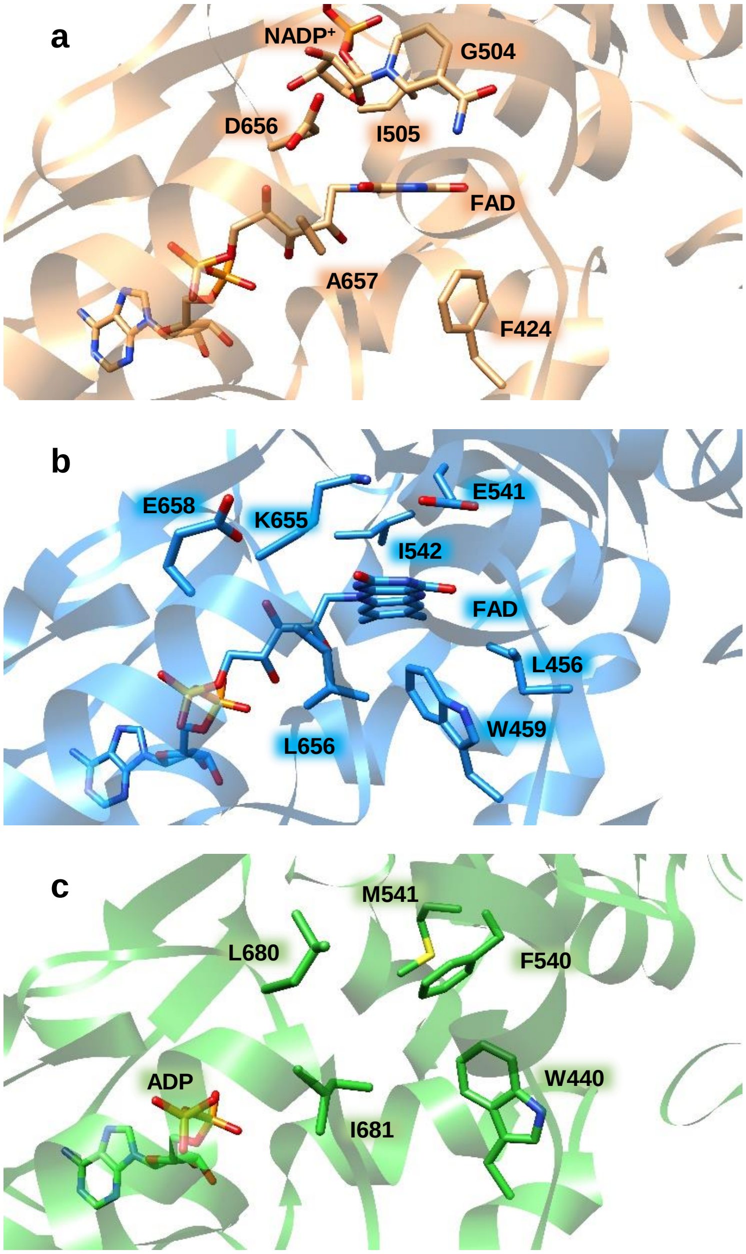

Supplementary Figure 1. **Aligned structures of the FAD/ADP binding sites in NCR and related enzymes.** (a) 2,4-Dienoyl-CoA reductase in complex with $NADP^+$ localized at the *re*-side of FAD. (b) In NCR, the more bulky W459 replaces F424 in DCR that pushes the FAD from the *si*-side upwards thereby destroying the NADPH binding site. (c) Trimethylamine dehydrogenase also contain the bulky tryptophane (W440) partly filling the gap because FAD is replaced by a redox inactive ADP for retaining structural integrity.

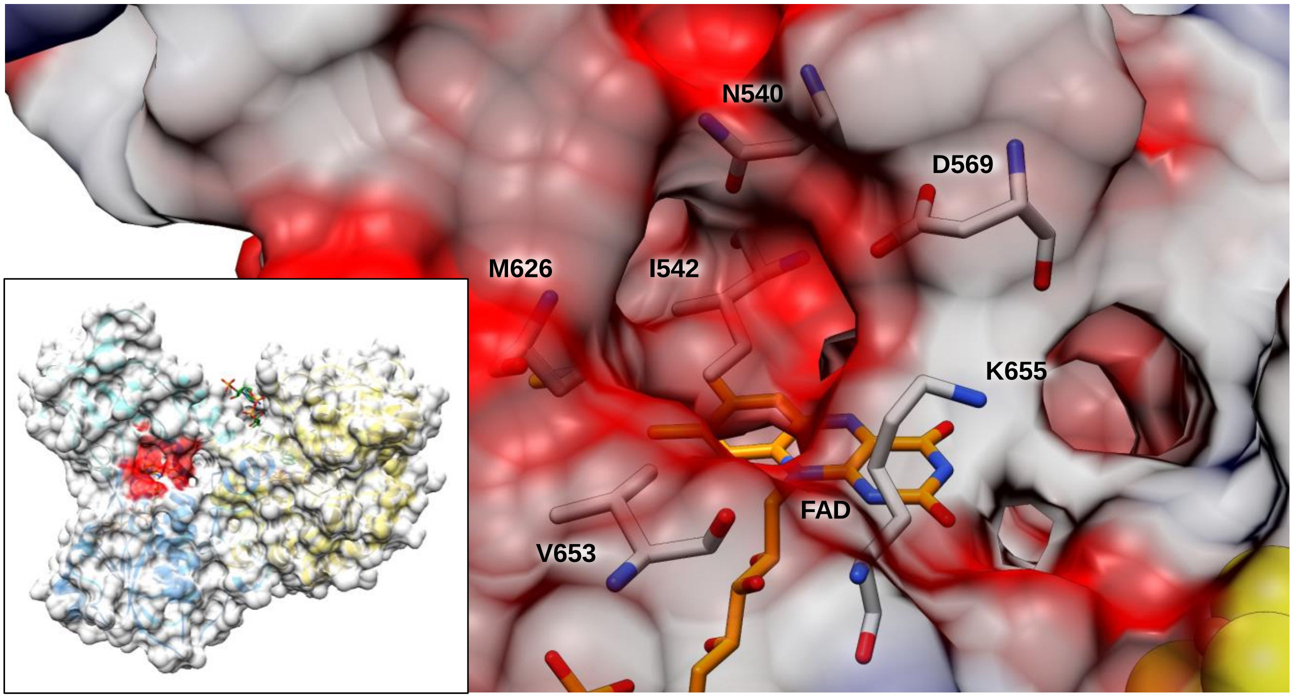

Supplementary Figure 2. Potential electron donor interaction site of NCR. The unknown, most possibly proteinogenic, electron donor presumably binds into a flat hollow with its redox cofactor in an electron-conducting distance to FAD. Its binding site is formed by the depicted amino acids. The polypeptide region around FAD is characterized by a rather negative electrostatic potential. The surface is gradually colored by the assigned Coulomb charges from most positive (blue) to most negative (red).

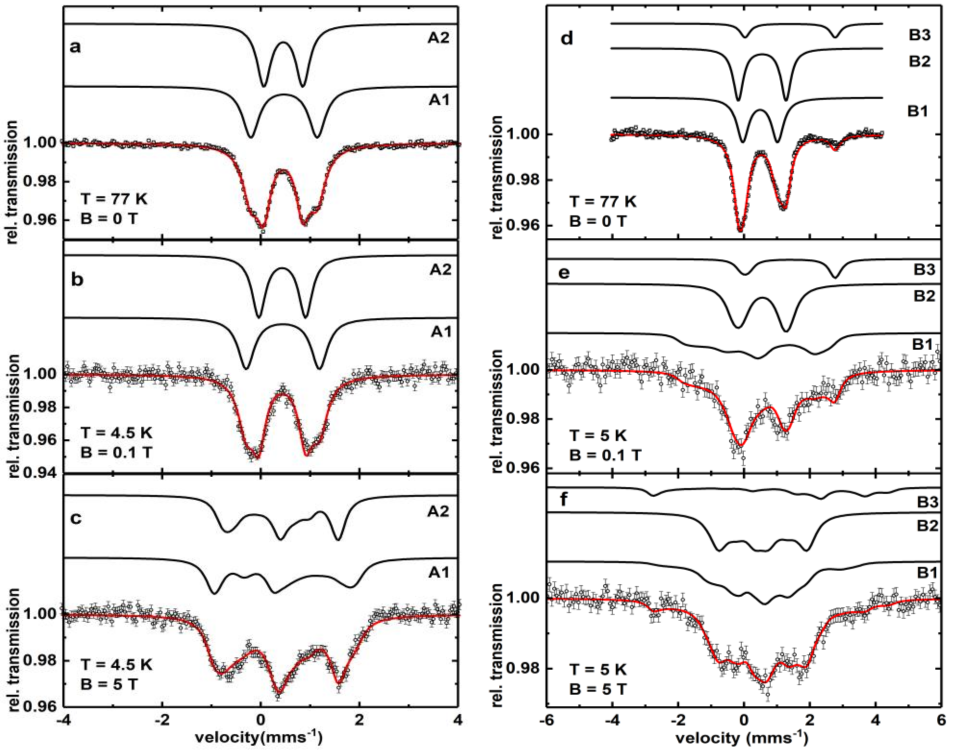

Supplementary Figure 3. Mössbauer spectra of NCR. The simulations (red solid line) representing the sum of the subcomponents (black lines) and the measured data points with deviation are shown. Oxidized NCR recorded at T = 77 K without an external field (a) and at T = 4.5 K in a field of B = 0.1 T (b) and B = 5 T (c). Component A1 and A2 are simulated in a ratio of 1:1. For the analysis a $[4Fe\text{-}4S]^{2+}$ cluster in a diamagnetic S = 0 state was assumed. The Mössbauer parameters are listed in Supplementary Table 2. Dithionite reduced NCR recorded at T = 77 K without an external field (d) and at T = 5 K in a field of B = 0.1 T (e) and B = 5 T (f). For component B1 and B2 a $[4Fe\text{-}4S]^{1+}$ cluster in a paramagnetic S = ½ state with g = (2.0; 2.0; 2.0) was assumed. Component B3 was assigned to Fe^{2+} in a paramagnetic S = 2 state. The Mössbauer parameters are listed in Supplementary Tables 2 and 3. Source data are provided as a Source Data file

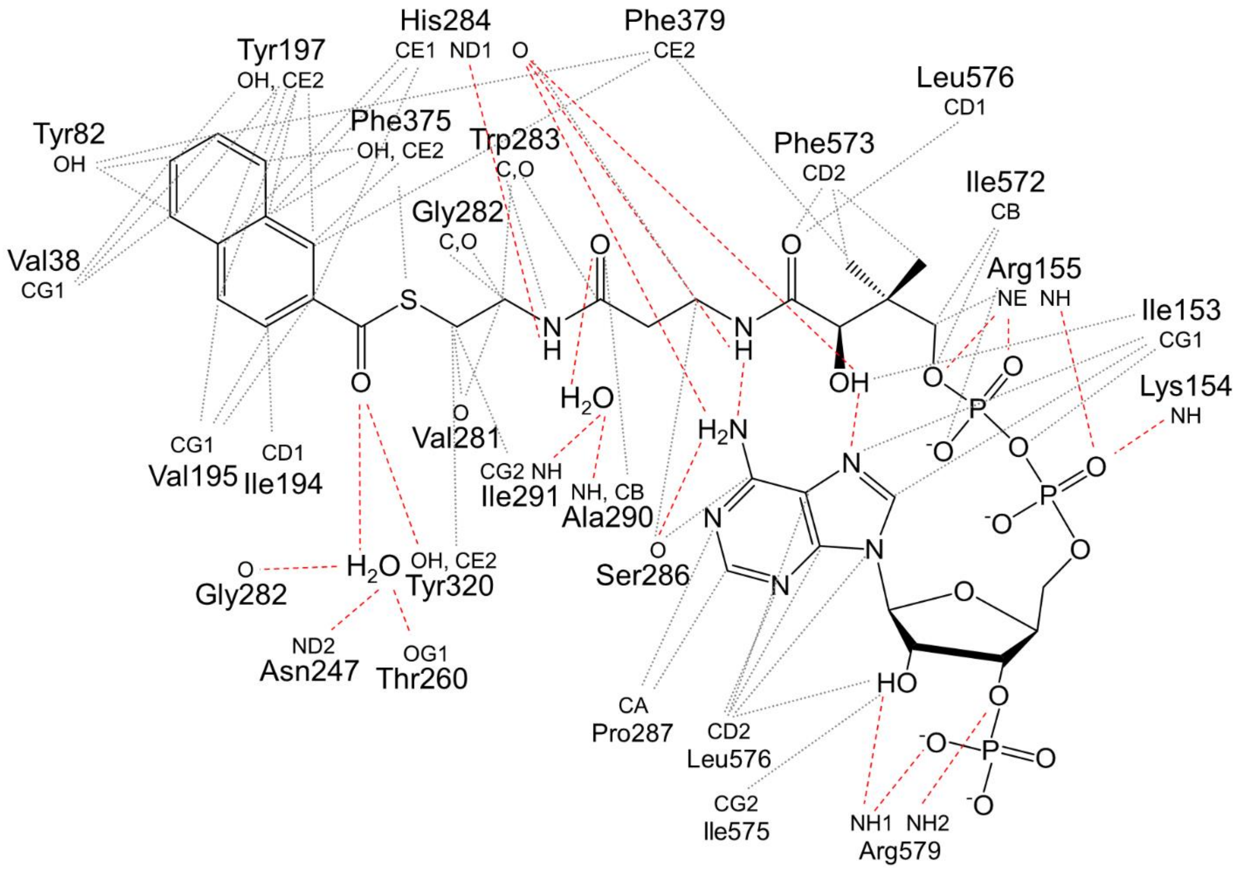

Supplementary Figure 4. Scheme of the naphthoyl-CoA – polypeptide contacts. Van-der-Waals and hydrogen bond interactions are drawn up to a distance of 4 Å and to 3.5 Å in gray and red dashed lines, respectively. Every naphthalene atom forms, in addition, one or more Van-der-Waals contacts to the isoalloxazine ring which are omitted for reasons of clarity. Hydrogen bond but not Van-der-Waals interactions between the adenine ring and the pantetheine moieties of CoA are shown.

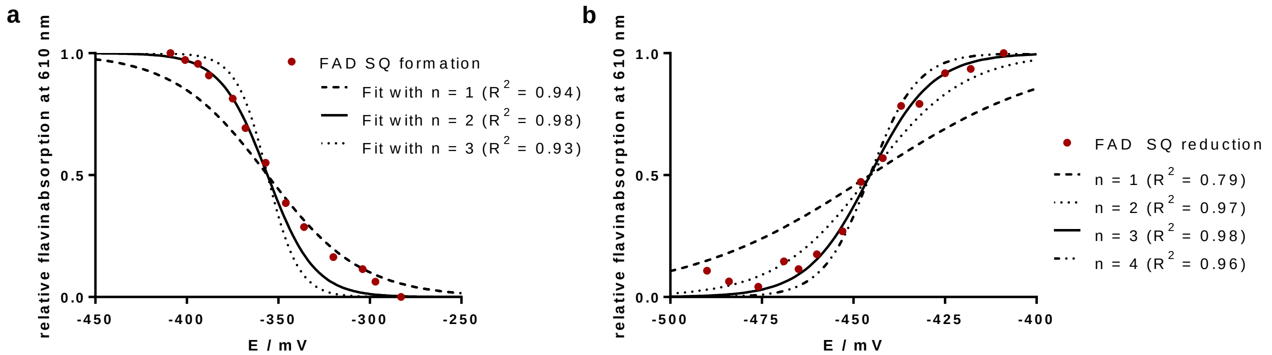

Supplementary Figure 5. Fit of Nernst-Curves with varying n-values to experimental data obtained during redox titrations. a, Reduction of FAD to FADH· fitted to Nernst curves with n = 1 (dashed line) and n = 2 (best fit, solid line) and n = 3 (dotted line) electrons. **b,** Reduction of FADH· to $FADH^-$ fitted to Nernst curves with n = 1 (dashed line), n = 2 (dotted line), n = 3 (best fit, solid line) and n = 4 (dashed/dotted line) electrons. The red dots represent measured data points from the redox titrations. Source data are provided as a Source Data file.

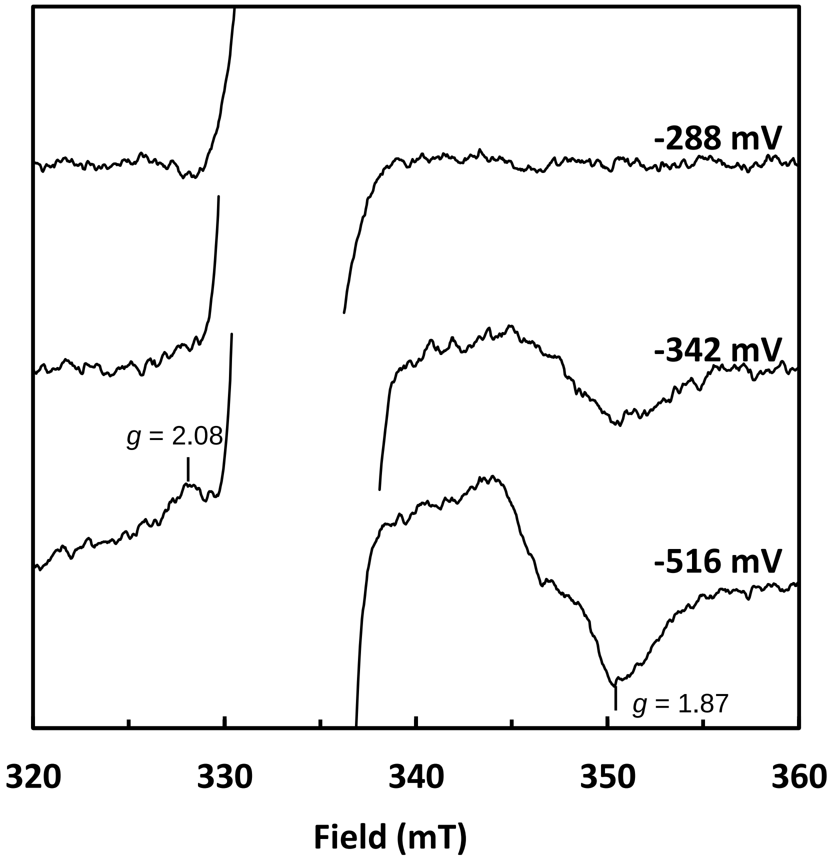

Supplementary Figure 6. Representative EPR spectra of NCR samples. The poised redox potentials during dye-mediated redox titration are indicated. The strong radical signal ranging from 330-337 mT derived from the redox dyes, and, in the E = –342 mV sample from the FAD SQ, has been truncated for better presentation. Note that in the E = –342 mV sample the $[4Fe\text{-}4S]^{+1}$ cluster S=1/2 signal is broadened compared to the fully developed one at E = –516 mV. This finding is assigned to a strong magnetic coupling between the $[4Fe\text{-}4S]^{+1}$ cluster and the FAD SQ. The FAD SQ is present in the E = –342 mV, but is fully reduced in the E = –516 mV sample. EPR conditions: temperature, 10 K; microwave power, 20 mW; modulation amplitude, 1.5 mT; modulation frequency, 100 kHz. Source data are provided as a Source Data file.

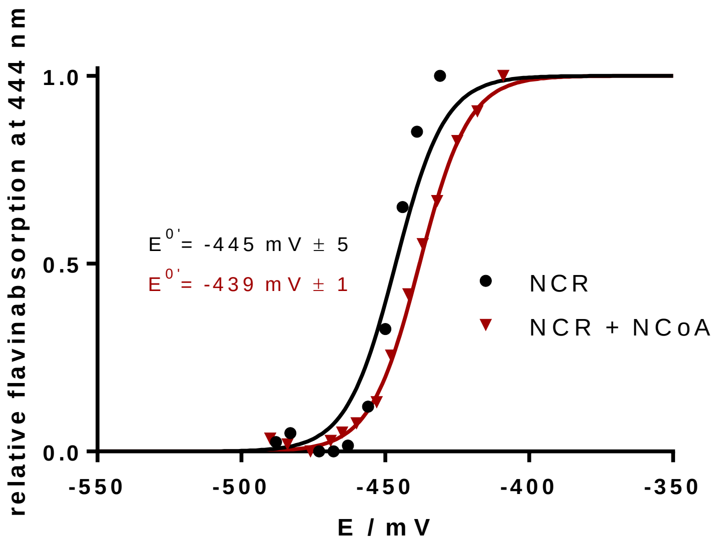

Supplementary Figure 7. Redox titration of the FMN cofactor. The absorption at 444 nm is plotted vs the redox potential during titration of NCR (20 µM) in the presence (red) and absence of NCoA (black). At potentials below –400 mV the FAD cofactor is fully reduced to the semiquinone, and the decrease of absorbance with decreasing redox potential can be fully assigned to the two-electron reduction of FMN to $FMNH^{-}$ without intermediary formation of an FMN semiquinone. The absorption at 444 nm was normalized and fitted to a Nernst-curve with n = 3 electrons as next to the two-electron reduction of FMN to $FMNH^{-}$ the concurrent reduction of FAD semiquinone to $FADH^{-}$ takes place at an almost identical redox potential. Source data are provided as a Source Data file.

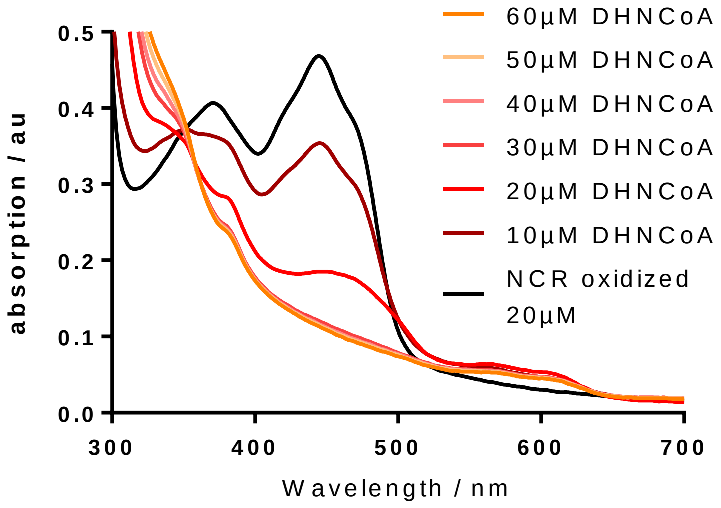

Supplementary Figure 8. UV/vis spectra of oxidized NCR during titration with DHNCoA. DHNCR was added stepwise to NCR (20 µM) at pH 8.0 as shown. The FAD semiquinone with characteristic absorptions at 566 and 610 nm cannot be reduced by an excess of the two-electron donor DHNCoA. Source data are provided as a Source Data file.

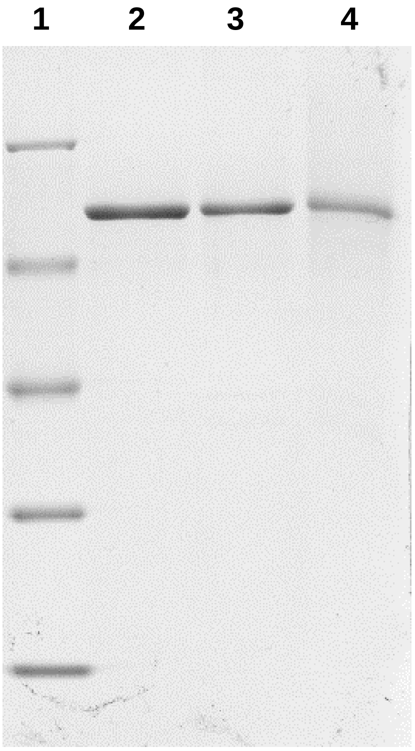

Supplementary Figure 9. SDS-PAGE analysis of NCR purification steps by column chromatography. Pierce™ unstained protein MW marker (Thermo Scientific, Karlsruhe, Germany) was applied to lane 1 as a reference. NCR was applied at 2 µg each after individual chromatography steps. Lane 2: after Superdex 200 PG gel filtration. Lane 3: after ResourceQ anion exchange chromatography. Lane 4: after streptactin affinity chromatography. Source data are provided as a Source Data file.

Supplementary Tables

Supplementary Table 1. Mössbauer parameters obtained from the simulation of the oxidized NCR spectrum (see Supplementary Figure S3)

	A1	A2
δ (mms^{-1})	0.45	0.43
ΔE_q (mms^{-1})	1.48 (1.31)	-0.95 (0.77)[1]
Γ (mms^{-1})	0.39	0.33
η	0	0
Area (%)	50	50

[1]The quadrupole splitting obtained from the simulation of the 77 K spectrum is displayed in brackets.

Supplementary Table 2. Mössbauer parameters obtained from the simulation of the dithionite-reduced NCR spectrum (see Supplementary Figure S3).

	B1	B2	B3
δ (mms-1)	0.49	0.55	1.40
ΔE_q (mms^{-1})	1.06	1.46	2.74
Γ (mms^{-1})	0.44	0.57	0.40
η	0.6	1	1
$A_{x,y,z}/g_N \mu_N$ (T)	(-28/-5.7/-13.7)	(2.3/-2.6/-1.2)	(-12.4/-12.8/-2.5)
D (cm^{-1})	-	-	20
Area (%)	44	44	12

Supplementary Table 3. Mediator compounds used for redox titration coupled to UV/vis spectroscopy.

Mediator	$E^{o'}$ /
2,2'-Bipyridin	-500
Methylviologen dichloride hydrate	-449
Benzylviologen dichloride	-358
Neutral Red	-325
Safranin T	-289
Anthraquinone-2-sulfonic acid sodium salt monohydı	-225
2-Methyl-1,4-naphthoquinone (Menadione)	-203
Indigo carmine disodium salt	-125

Supplementary Methods

UPLC analyses. Samples were separated by reversed-phase chromatography (Knauer C-18 Eurospher II 100-2 column, 2.0 x 100 mm, 2 µm particle size) on an Aquity H class UPLC (Waters, Milford, USA) using ACN/10 mM potassium phosphate pH 6.8 as eluent. Compounds eluted in a linear gradient of 15-35% ACN from 0 to 5.8 minutes and were detected by UV diode array and identified by comparison to UV spectra of standards.[1]

Structure depictions. Figures of the crystal structure were prepared by using UCSF Chimera 1.13.[2]

Mössbauer spectroscopy. Transmission Mössbauer spectra were recorded with a conventional Mössbauer spectrometer operated in the constant acceleration mode in conjunction with a multi-channel analyzer in the time-scale mode (WissEl GmbH). The spectrometer was calibrated against α-iron at room temperature. A flow cryostat (Optistat^{DN}, Oxford Instruments) was used to measure the samples at 77 K. Field-dependent conventional Mössbauer spectra were obtained with a helium closed-cycle cryostat (CRYO Industries of America, Inc.) equipped with a superconducting magnet.[3] The magnetic field was aligned parallel to the γ-ray beam. The spectral data were transferred from the multi-channel analyzer to a PC for further analysis employing the public domain program Vinda[4] running on an Excel 2003® platform. The spectra were analyzed by least-squares fits using Lorentzian line shapes with the linewidth Γ. Field-dependent spectra were simulated by means of the spin Hamilton formalism.[5] Samples of ^{57}Fe-labeled NCR were used at a concentration of 1.02 mM (81 mg/mL) and anaerobically mixed with freshly prepared sodium dithionite solution (500 mM, 13.8 mM final concentration) or solid DHNCoA (5 mM final concentration) to obtain fully reduced enzyme.

EPR spectroscopy.

EPR spectra were recorded with a Bruker Elexsys E580 EPR spectrometer and 4122HQE-W1/1017 resonator working in the perpendicular mode. The low-temperature measurements were realized with an Oxford Instruments ESR 900 helium continuous-flow cryostat. NCR was reduced by incubation with 2 mM sodium dithionite and oxidized by incubation with 2 mM potassium ferricyanide.

For the EPR-monitored dye-mediated redox titration in the anaerobic tent a final concentration of 25 µM NCR in buffer (50 mM HEPES/KOH pH 8.0, 150 mM KCl) was used. As dyes Safranin T (E = –289 mV), neutral red (E = –329 mV), benzyl viologen (E = –358 mV) and methyl viologen (E = –449 mV) at 40 µM concentration were employed. Solution potentials were measured with an InLab Redox Micro ARGENTHAL© electrode (METTLER TOLEDO) connected to Education line EL20 multimeter under anaerobic conditions. After 30 min equilibration small amounts of freshly prepared sodium dithionite in buffer were added. EPR samples were collected after stabilization of the potential, shock-frozen and stored in liquid nitrogen for EPR analysis. From a fit to the Nernst equation (n=1) of the normalized height of the derivative-shaped feature at g=1.926 the redox midpoint potential of the $[4Fe\text{-}4S]^{1+}$ cluster was determined.

Supplementary References

1. Estelmann, S., Blank, I., Feldmann, A., Boll, M. Two distinct old yellow enzymes are involved in naphthyl ring reduction during anaerobic naphthalene degradation. *Mol. Microbiol.* **95,** 162–172 (2015).
2. Pettersen, E. F. *et al.* UCSF Chimera-A visualization system for exploratory research and analysis. *J. Comput. Chem.* **25,** 1605–1612 (2004).
3. Janoschka, A., Svenconis, G. & Schünemann, V. A closed cycle-cryostat for high-field Mössbauer spectroscopy. *Journal of Physics: Conference Series* **217,** 012005 (2010).
4. Gunnlaugsson, H. P. Spreadsheet based analysis of Mössbauer spectra. *Hyperfine Interact.* **237,** 79 (2016).
5. Schünemann, V. & Winkler, H. Structure and dynamics of biomolecules studied by Mossbauer spectroscopy. *Rep. Prog. Phys.* **63,** 263–353 (2000).
